# A Splicing Mutation in *mitfa* is Involved in the Depigmentation of Cavefish *Triplophysa rosa*

**DOI:** 10.1093/molbev/msaf175

**Published:** 2025-07-25

**Authors:** Mingming Zhang, Qingshuo Zhao, Jinqing Huang, Ming Zou, Baocheng Guo, Yahui Zhao, Fanwei Meng

**Affiliations:** State Key Laboratory of Animal Biodiversity Conservation and Integrated Pest Management, and Key Laboratory of Zoological Systematics and Evolution, Institute of Zoology, Chinese Academy of Science, Beijing 100101, China; State Key Laboratory of Animal Biodiversity Conservation and Integrated Pest Management, and Key Laboratory of Zoological Systematics and Evolution, Institute of Zoology, Chinese Academy of Science, Beijing 100101, China; School of Basic Medical Sciences, Guilin Medical University, Guilin, Guangxi 541199, China; School of Nursing and Health Management, Wuhan Donghu College, Wuhan, Hubei 430212, China; State Key Laboratory of Animal Biodiversity Conservation and Integrated Pest Management, and Key Laboratory of Zoological Systematics and Evolution, Institute of Zoology, Chinese Academy of Science, Beijing 100101, China; State Key Laboratory of Animal Biodiversity Conservation and Integrated Pest Management, and Key Laboratory of Zoological Systematics and Evolution, Institute of Zoology, Chinese Academy of Science, Beijing 100101, China; State Key Laboratory of Animal Biodiversity Conservation and Integrated Pest Management, and Key Laboratory of Zoological Systematics and Evolution, Institute of Zoology, Chinese Academy of Science, Beijing 100101, China

**Keywords:** cavefish, *Triplophysa rosa*, pigmentation, transcriptome, *mitfa*, alternative splicing

## Abstract

Regression traits such as pigmentation loss in cave-dwelling species offer powerful models for understanding evolutionary mechanisms under extreme environments. In this study, we investigated the genetic and evolutionary mechanisms underlying pigmentation loss in the cavefish *Triplophysa rosa*, a depigmented, eyeless species endemic to subterranean habitats. Compared with its surface-dwelling relative *T. stenura*, *T. rosa* exhibited significantly reduced expression of melanogenesis genes, indicating transcriptional repression of pigmentation pathways. Further analysis revealed a novel splicing site mutation in *melanocyte inducing transcription factor a* (*mitfa*), which results in a 63-nt deletion and loss of 21 amino acids in the activation domain. Functional rescue assays in zebrafish confirmed that the loss of 21 amino acids in Mitfa severely compromises melanin synthesis. Additionally, a premature stop codon in *tyrosinase-related protein 1a* (*tyrp1a*) was detected, which may also contribute to the depigmented phenotype. Evolutionary analyses indicated that pigmentation-specific genes in the *T. rosa* lineage are under relaxed purifying selection, consistent with weakened selective constraints on pigmentation in cave environments. Collectively, our findings indicate that a splice-site mutation in *mitfa*, acting against a background of relaxed selection on pigmentation genes, contributes to pigmentation loss in *T. rosa*, offering integrated proximate (molecular) and evolutionary insights into the troglomorphic traits in cavefish.

## Introduction

Understanding the genetic and evolutionary mechanisms driving trait regression, such as pigmentation loss, remains a fundamental challenge in evolutionary biology (Jeffery 2009). Trait regression, especially in organisms inhabiting unique and extreme environments like caves, raises questions about the contributions of neutral mutations, relaxed purifying selection, and adaptive processes in shaping phenotypic outcomes (Wilkens 1988; Rétaux and Casane 2013). The recurrence of regressive traits across diverse cave environments indicates a pattern of convergent evolution, and cavefish serve as an ideal model for investigating these evolutionary processes.

Cavefish exhibit highly specialized traits to perpetual darkness, including pigmentation loss, eye degeneration, enhanced nonvisual sensory systems, circadian clock dysregulation, and the loss of schooling behavior (Yoshizawa et al. 2012; Kowalko et al. 2013; Meng et al. 2013; McGaugh et al. 2014; Mack et al. 2021; Blin et al. 2024). Cavefish inhabit karst cave water year round (subterranean waterbodies), completing much or all of their life cycle in darkness (Zhao et al. 2021). Among over 350 known cave species worldwide, China harbors the highest diversity, with 148 valid species accounting for 39% of the global stygobiontic fish diversity (Ma et al. 2019; Zhao et al. 2022b; Jiang et al. 2023). More than half of these Chinese cavefishes are stygobionts that have adapted to subterranean habitats (Zhao et al. 2022b). These cavefish resources offer valuable opportunities to investigate the genetic mechanisms and evolutionary dynamics driving such evolution.

Fish skin coloration and pigment patterns arise from the neural crest-derived chromatophores, including melanophores, xanthophores, iridophores, erythrophores, cyanophores, and leucophores (Nüsslein-Volhard and Singh 2017). Skin pigmentation within melanophores is primarily determined by melanin synthesis, which protects against UV radiation and contributes to camouflage and signaling (del Marmol and Beermann 1996; Bian et al. 2021). Unlike mammals and birds, where eumelanin and pheomelanin coexist, only eumelanin has so far been observed in fish (Burton 2011).

Teleost-specific genome duplication has increased the number of pigmentation genes compared with tetrapods, contributing to the complexity of pigmentation pathways in fish (Braasch et al. 2009). To date, over 125 pigmentation genes have been identified as critical for pigmentation in fish (Parichy 2006; Li et al. 2020). Numerous studies have revealed that mutations, especially loss-of-function (LoF) variants, in pigmentation genes are linked to reduced pigmentation or complete pigment loss across diverse taxa (Braasch et al. 2007; Policarpo et al. 2021). For example, in *Astyanax mexicanus*, albinism and depigmentation in cavefish are linked to *oculocutaneous albinism type 2* (*oca2*) and *melanocortin receptor 1* (*mc1r*), respectively (Protas et al. 2006; Gross et al. 2009; Stahl and Gross 2015; Klaassen et al. 2018). Besides LoF coding mutations, upstream cis-regulatory region variation in *mc1r* has been shown to cause the regressive pigmentation in cave populations of *A. mexicanus* (Stahl and Gross 2015). In contrast, genomic studies of *Sinocyclocheilus* cavefish have revealed no deletions in *oca2* and *mc1r*, but instead a conserved mutation in *tyrosinase* (*tyr*). In addition, *tyrp1*, *gnaq*, *nras*, and *p38* are significantly downregulated in cavefish relative to their surface relatives (Yang et al. 2016; Li et al. 2020). Other LoF mutations in pigmentation-related genes have been identified across various cave-dwelling species, including *tyrp1a* and *premelanosome protein b* (*pmelb*) in *S. anshuiensis* cavefish, *pmelb* in the albino deep-sea snailfish (*Pseudoliparis swirei*), and *solute carrier family 24 member 5* (*slc24a5*) in cave-restricted *A. mexicanus* (Bian et al. 2021). Additionally, convergent gene decay has been observed in pigmentation genes in multiple cavefish lineages, exemplified by shared LoF mutations in *adamts20* in *Lucifuga dentata* and *L. gibarensis*, as well as independent pseudogenization events in *GTP cyclohydrolase 2* (*gch2*) and *pmelb* in both blind and small-eyed *Sinocyclocheilus* species, highlighting the repeated and convergent nature of gene decay in pigmentation pathways among cavefishes (Policarpo et al. 2021). *Mitf* directly or indirectly regulates the expression of over 40 pigmentation genes, including *dopachrome tautomerase* (*dct*), *oca2*, *slc24a5*, and members of the tyrosinase family (*tyr*, *tyrp1a*, and *tyrp1b*), establishing its role as the master regulator of melanocyte development and melanogenesis (Cheli et al. 2010; Vachtenheim and Borovanský 2010; Kawakami and Fisher 2017; Goding and Arnheiter 2019). Teleost possess *mitfa* and *mitfb*, which respectively function in epidermal melanocytes (*mitfa*) and the retinal pigment epithelium (RPE) (*mitfb*) (Lister et al. 2001). Mutations in *mitfa* lead to complete albinism in both the zebrafish *nacre* mutant and the transparent icefish (*Protosalanx hyalocranius*) (Lister et al. 1999; Bian et al. 2021). However, the genetic and evolutionary mechanisms driving pigmentation loss in cavefish remain incompletely resolved and warrant further investigation.

Pigmentation loss in cavefish could result from a complex interplay of evolutionary mechanisms, including relaxed purifying selection, adaptive pressures, and gene flow (Gross et al. 2016; Jeffery et al. 2016; Herman et al. 2018; Moran et al. 2023). In the absence of light, relaxed purifying selection can render deleterious mutations effectively neutral, allowing them to reach fixation by genetic drift, especially in pigmentation-specific genes that are primarily involved in the pigmentation process. At the same time, natural selection may also play a role by favoring energy conservation or other adaptive benefits in the lightless environment. Gene flow adds another layer of complexity by facilitating the transfer of genomic regions associated with troglomorphic traits, including pigmentation loss, between cave populations (Policarpo et al. 2024). Although these mechanisms are not mutually exclusive, evidence for their relative contributions remains inconclusive.

Alternative splicing can be involved in phenotype evolution, as mutations in splice sites can result in the generation of isoforms with novel exon combinations, enabling rapid evolutionary innovation (Wright et al. 2022). For example, in three spine sticklebacks, the use of an alternative 5′ splice site in the first exon of *muscle segment homeobox 2a* (*msx2a*) generates a shortened, nonfunctional transcript with increased expression, which contributes to their shorter dorsal spines in freshwater populations (Howes et al. 2017). Moreover, alternative splicing has been demonstrated to be crucial for regulating growth, development, and environmental adaptation in fish (Tian et al. 2020; Xiao and Wang 2022). A study on cichlids has also shown that the rapid evolution in explosively speciated lineage is facilitated by the acquisition of complex regulatory mechanisms for alternative splicing over a very short period (Terai et al. 2003). However, the role of alternative splicing in cavefish albinism has not yet been well characterized in a natural system.

*Triplophysa* is one of largest genera in *Nemacheilidae* (Cypriniformes), with 147 identified species primarily distributed in and adjacent to the Qinghai–Xizang (Tibet) Plateau. Although most *Triplophysa* species inhabit mountain streams and lakes, at least 27 species are cave-dwelling (Zhao et al. 2022a). Recent mitogenomic analyses have resolved the genus into four well-supported clades (I–IV), with all known cave-dwelling species forming a monophyletic group within Clade IV (Wang et al. 2023b). *T. stenura* (a surface loach with typical pigmentation and normal eyes) and *T. rosa* (an eyeless and depigmented cave-dweller) are classified in different clades, indicating distinct evolutionary lineages. Previous studies have estimated that the *Triplophysa* lineage diverged from other loaches ∼23.5 million years ago (95% CI: 20.5 to 26.1 Ma), with the clade containing *T. rosa* likely originating around 21.3 Ma (95% CI: 18.2 to 24.1 Ma) (Wang et al. 2016).

This study explores the genetic and molecular mechanisms underlying depigmentation in *Triplophysa* cavefish by examining the cave-dwelling species *T. rosa* alongside the surface species *T. stenura*. Our histological analyses first confirmed the depigmented phenotype in *T. rosa*. Comparative transcriptome profiling and quantitative PCR further revealed the substantial down-regulation or absence of genes involved in melanin synthesis in this cavefish. Additionally, we identified a splicing mutation in *mitfa*, which indicates it as a critical genetic contributor to depigmentation in the cavefish *T. rosa*, and this finding was validated by functional assays. Moreover, a premature stop codon mutation identified in *tyrp1a* also likely contributes to pigmentation loss in this species. Evolutionary analyses of Ka/Ks ratios revealed that pigmentation-specific genes in *T. rosa* lineage exhibited signs of relaxed purifying selection compared with those in surface fish lineage, consistent with reduced evolutionary constraints in the cave environment. Together, these findings advance our understanding of the transcriptional regulation and molecular changes driving depigmentation, offering broader insights into the evolution of troglomorphic traits in cavefish.

## Results

### Depigmentation of Skin Melanophores in the Cavefish *T. rosa*

**Fig. 1.**
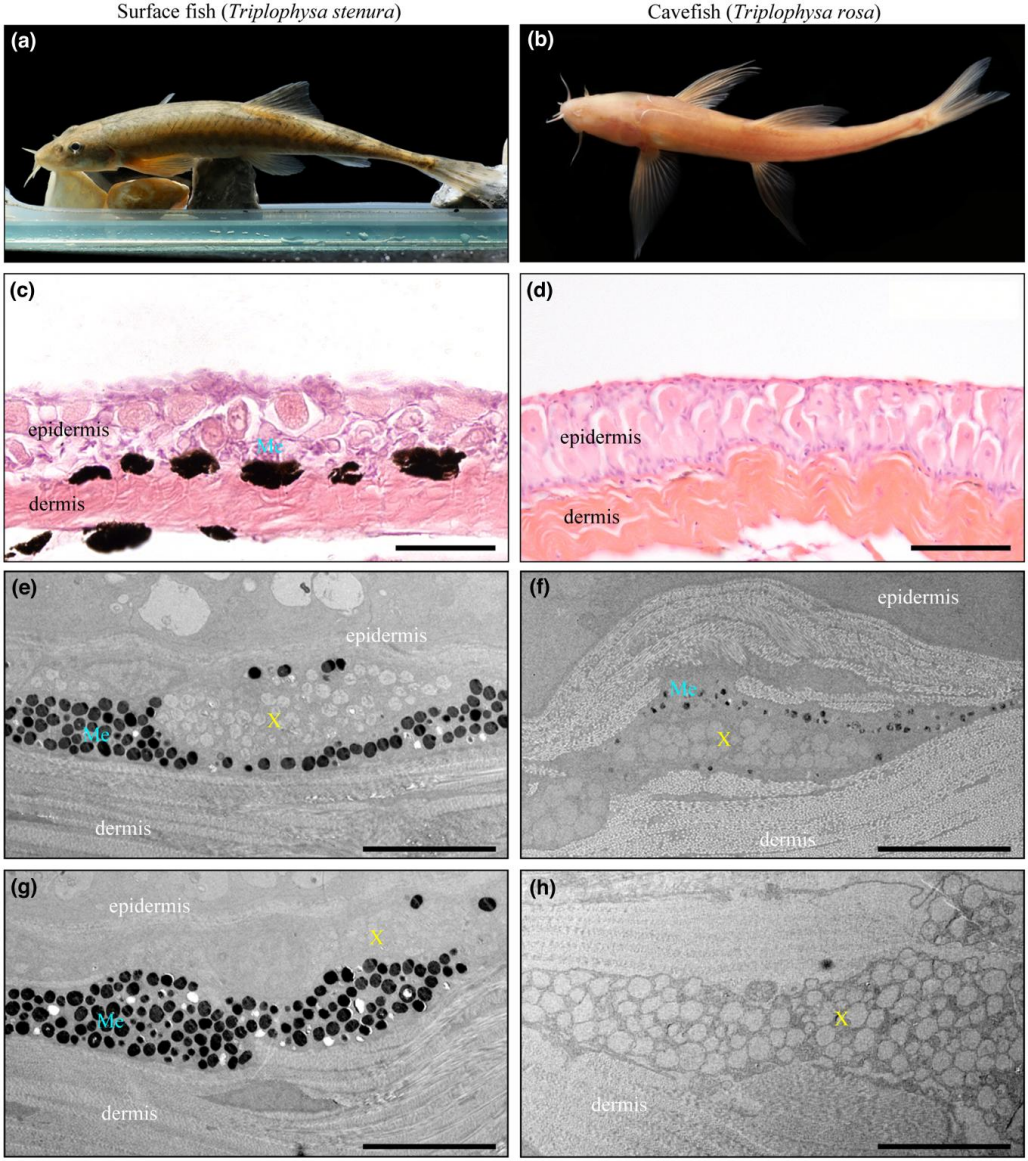
a) Photograph of a surface fish, *T. stenura*. b) Photograph of a cavefish, *T. rosa*. c, d) Hematoxylin and eosin (H&E) stained sections of the skin from a surface fish c) and a cavefish d). e–h) Transmission electron micrographs of melanophore in the skin of adult *T. stenura* (e, g) and *T. rosa* (f, h). Me, melanophores; X, xanthophores. Scale bars in (c, d), 50 μm; (e–h), 5 μm.

Living in perpetual darkness, cavefish often exhibit a depigmented or albino appearance. Observations revealed that the skin of the cave species *T. rosa* was slightly pink, likely due to red blood cells showing through the skin, in contrast to the normally pigmented surface species *T. stenura* (Fig. 1a and b). Through hematoxylin and eosin (H&E) staining and transmission electron microscopy, we found there were fewer melanosomes in the skin of cavefish compared with surface fish (Fig. 1c and d). In addition, most cavefish skin appeared to lack visible melanosomes or contained only lightly pigmented melanosomes (Fig. 1e–h).

### Relaxed Selection on Pigmentation Genes in the Cavefish *T. rosa*

We sequenced the skin transcriptomes of the two *Triplophysa* species using the Illumina Hiseq 2000 platform, generating 269,042,942 clean reads and 40.36 Gb bases. Sequencing data were deposited in the China National Center for Bioinformation database under Genome Sequence Archive accession number CRA006704. Read and base numbers, GC content, and other quality parameters are presented in supplementary table S1, Supplementary Material online.

**Fig. 2.**
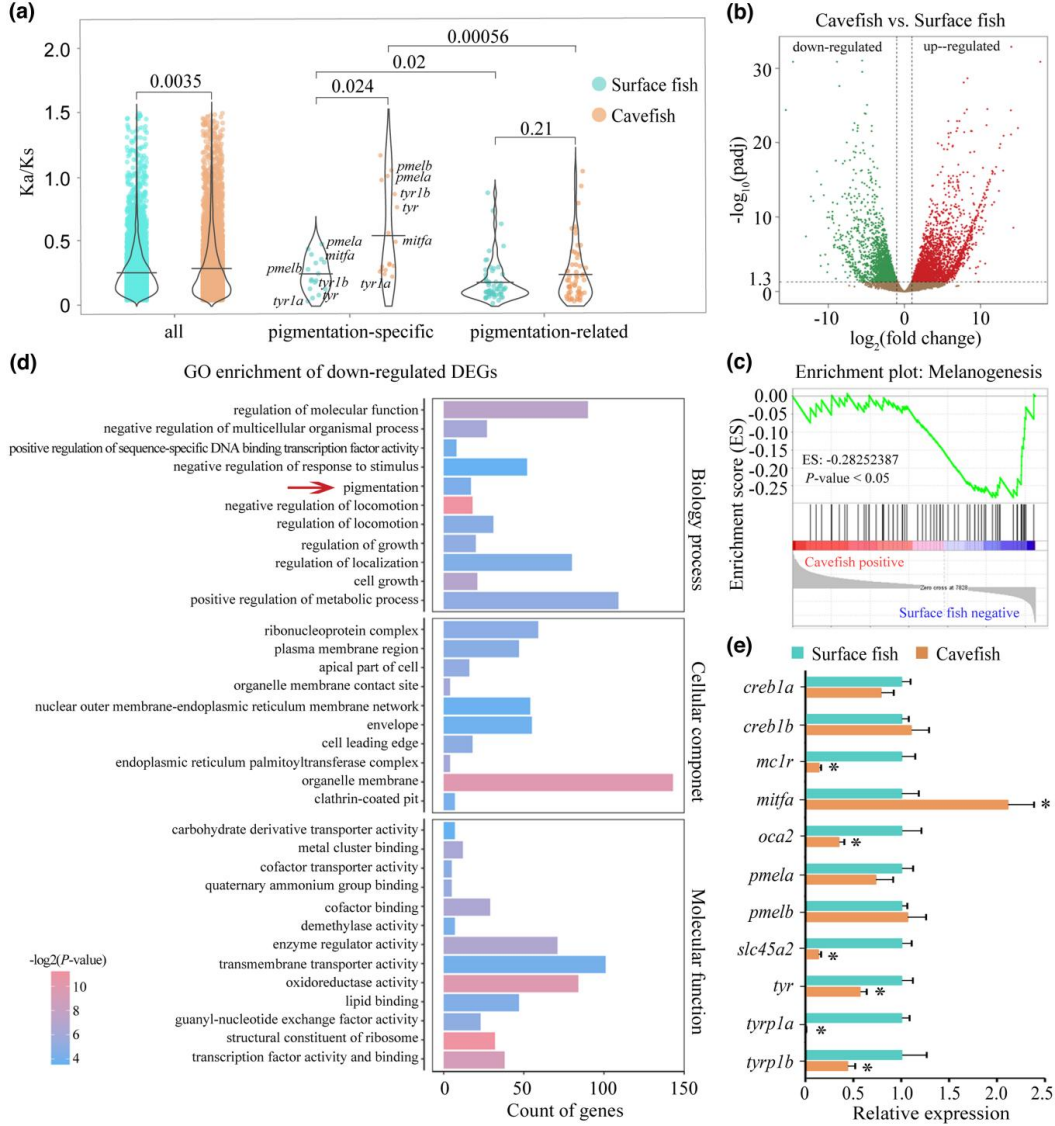
a) Violin plot showing the distribution of Ka/Ks ratio (*ω*) of all orthologs, “pigmentation-specific” genes, and “pigmentation-related” genes in surface fish and cavefish. Horizontal lines within each violin represent the mean *ω* values for the gene category in each species. *P*-values indicating statistical significance between groups are displayed above the corresponding horizontal bars. b) Volcano plot displaying the distribution of DEGs in the skin between surface and cavefish. Upregulated DEGs are indicated by red dots (right panel), downregulated DEGs by green dots (left panel). DEGs were defined with an adjusted *P* < 0.05 (−log_10_(*P*adj) > 1.3). c) GSEA analysis reveals that melanogenesis process had a less positive expression in cavefish compared with surface fish (*P* < 0.05). d) GO enrichment analysis of down-regulated DEGs in cavefish in three main categories. The *x*-axis indicates the number of genes associated with each term, and the *y*-axis lists the GO terms. Color intensity represents the statistical significance. The red arrow highlights the pigmentation term (GO:0043473). e) Quantitative real-time PCR validation of the core genes in the melanin synthesis pathway. Gene expression levels were analyzed using cDNAs derived from the skin of surface fish and cavefish, respectively. Data are presented as mean ± SEM. An asterisk (*) indicates that *P* < 0.05.

To investigate genome-wide selection pressures in the cavefish *T. rosa*, we identified 9,986 orthologous genes among *T. rosa*, *T. stenura*, and the outgroup species *Danio rerio* using PosiGene (Sahm et al. 2017). We then applied the branch model in PAML (Yang 2007) to estimate Ka/Ks ratios (*ω*) for each gene along the *T. rosa* and *T. stenura* lineages, using *D. rerio* as the outgroup. After calculating the Ka/Ks ratios, we generated Ka/Ks distribution plots for all orthologs and for the two candidate gene sets, “pigmentation-specific” and “pigmentation-related”, to explore lineage-specific selection patterns (Fig. 2a). We first constructed a comprehensive pigmentation gene set based on functional annotations in *D. rerio* (see Materials and Methods). Pigmentation-specific genes (*n* = 21) are primarily involved in melanin synthesis, melanocyte differentiation, or melanosome formation, while pigmentation-related genes (*n* = 94) have broader biological functions and can regulate pigmentation either directly or indirectly (supplementary table S2, Supplementary Material online). From the orthologous gene set, we retrieved 17 pigmentation-specific genes and 58 pigmentation-related genes (supplementary table S3, Supplementary Material online).

At the genome-wide level, the average *ω* in the cavefish (0.292) was slightly higher than in the surface fish (0.259). Notably, pigmentation-specific genes exhibited substantially higher *ω* in cavefish (0.547) then in surface fish (0.251), indicating relaxed purifying selection in the cavefish lineage. In contrast, pigmentation-related genes, which often have pleiotropic functions, showed no significant difference between the two species (cavefish: 0.246; surface fish: 0.186) (Fig. 2a).

Several pigmentation genes, notably *pmela*, *pmelb*, *tyr*, and *tyrp1b*, exhibited Ka/Ks ratios close to 1 in *T. rosa* (Fig. 2a). *Tyr* and *tyrp1b* encode the major enzymes in the melanin synthesis, while *pmela* and *pmelb* encode fibrillar scaffolds essential for stage II melanosome maturation (Bissig et al. 2016; Varghese et al. 2021). The elevated Ka/Ks ratios in these genes, along with similar trends observed in others (supplementary table S3, Supplementary Material online), suggest that these genes may be evolving under relaxed functional constraint, potentially approaching a nearly neutral regime. Overall, these results support the hypothesis that pigmentation-specific genes in cavefish have experienced relaxed purifying selection or nearly neutral evolution, while multifunctional pigmentation-related genes generally remain under strong purifying selection across lineages.

To estimate the timing of relaxed selection on pigmentation-specific genes in *T. rosa*, we applied the mixed-branch model (Meredith et al. 2009; Policarpo et al. 2021). We concatenated these gene sequences (23,619 nt) to obtain a more reliable estimate of *ω*, which was estimated to be 0.215 in the lineage leading to the surface fish and 0.413 in the lineage leading to the cavefish. By assigning these values respectively to functional and mixed branches, the time since pigmentation-specific genes entered a nearly neutral evolutionary regime was estimated to be ∼5.37 Ma (supplementary fig. S1, Supplementary Material online), corresponding to 25.2% of the divergence time of the *T. rosa* lineage (21.3 Ma), though this estimate assumes complete neutrality and may represent an upper bound.

### Gene Expression Changes Associated With Cavefish Depigmentation

We calculated TPM (transcripts per million reads) values for each gene, and a Pearson correlation heatmap of TPM values between samples showed high consistency in expression patterns among replicates from the same species; this confirmed the reliability of sample processing and data analysis (supplementary fig. S2, Supplementary Material online). Compared with surface fish, we identified 5,228 differentially expressed genes (DEGs) out of the 20,686 genes expressed in cavefish skin, including 2,865 up-regulated and 2,363 down-regulated genes (Fig. 2b). Gene set enrichment analysis (GSEA) indicated a significant reduction in the melanogenesis process in cavefish (Fig. 2c), which indicated that the down-regulation of melanogenesis-related genes may play a key role in the depigmentation of cavefish skin.

The gene ontology (GO) enrichment analysis revealed significant enrichment (*P* = 0.033) of the pigmentation GO term (GO:0043473) in the down-regulated genes of cavefish relative to surface fish (Fig. 2d); this was consistent with observed pigmentation degeneration in cavefish. Additionally, most down-regulated DEGs in cavefish were categorized under seven GO subcategories: “regulation of molecular function,” “regulation of localization,” “positive regulation of metabolic process,” “organelle membrane,” “enzyme regulator activity,” “transmembrane transporter activity,” and “oxidoreductase activity” (Fig. 2d). Moreover, several GO terms related to “response to stress,” “anatomical structure morphogenesis,” and “hydrolase activity” were significantly enriched in the up-regulated DEGs in cavefish (supplementary fig. S3, Supplementary Material online); this could reflect genes involved in the morphological and physiological adaptations specific to cave environments.

### Expression and Sequence Changes of Pigmentation Genes in Cavefish

To investigate the molecular mechanisms underlying depigmentation in cavefish, we first surveyed all 115 pigmentation genes in our RNA-seq data and then validated the RNA-seq results with real-time quantitative PCR (qPCR) of several genes (Fig. 2e). Among the 20 detected pigmentation-specific genes, five (*mc1r*, *oca2*, *slc45a2*, *tyrp1a*, and *tyrp1b*) were significantly down-regulated in cavefish, and none showed up-regulation (supplementary table S4, Supplementary Material online). *Dct* was excluded from further analysis due to negligible expression in both *Triplophysa* species. Although RNA-seq indicated that *tyr* expression in cavefish was ∼61.8% of that in surface fish, qPCR confirmed its significant down-regulation (Fig. 2e). Reduced expression of *tyr*, *tyrp1a*, and *tyrp1b* likely severely impairs melanin synthesis. Additionally, down-regulation of *slc45a2* and *oca2* might compromise the melanosome microenvironment, indirectly reducing tyrosinase activity and melanin synthesis efficiency. The reduced expression of *mc1r*, which plays a central role in regulating melanin synthesis by promoting eumelanin production and inhibiting pheomelanin (Le Pape et al. 2008), might further attenuate melanin synthesis in the cave environment. Among the 77 detected pigmentation-related genes, 11 were down-regulated (*adrb2a*, *dio2*, *ednrba*, *gfpt1*, *inpp5b*, *rab27b*, *sox5*, *sox9b*, *tfap2e*, *trim33*, *ttc8*), and seven were up-regulated (*arl6*, *asip1*, *bbs2*, *bbs4*, *gart*, *paics*, *prdm1a*). Down-regulation of *ednrba* and *rab27b* may reduce melanosome transport and melanoblast migration (Fukuda 2005; Patterson and Parichy 2013). As an antagonist of *mc1r*, upregulation of *asip1* enhances its inhibitory effect on *Mc1r* activity, thereby suppressing melanin synthesis in line with depigmentation (Suzuki et al. 1997; Hida et al. 2009). The up-regulation of *bbs2*, *bbs4*, and *arl6*, which are involved in melanosome maturation and transport (Yen et al. 2006; Pretorius et al. 2010), may reflect compensatory mechanisms to maintain residual melanosome trafficking, although these may also have non-pigmentary roles in other skin cell types.

Our results showed that *pmela*, and *pmelb* exhibited stable expression in cavefish skin (supplementary table S4, Supplementary Material online and Fig. 2e). PMEL is a key protein in the formation of amyloid fibrils in melanosomes, which provide essential scaffolds for melanin synthesis (Bissig et al. 2016). The comparable expression of *pmela* and *pmelb* between surface fish and cavefish indicates that cavefish skin possesses melanocytes and melanosomes, which may still serve as a scaffold for melanin synthesis. We observed significant down-regulation of multiple genes directly involved in melanin synthesis, including *oca2*, *slc45a2*, *tyr*, *tyrp1a*, and *tyrp1b*, in cavefish (Fig. 2e). Because *mitfa* is the master transcription factor controlling these downstream targets, we anticipated a corresponding decrease in *mitfa* expression. Notably, contrary to expectations, both qPCR and RNA-seq (fold change = 2.74, *P* = 0.1697) revealed *mitfa* was highly up-regulated in cavefish compared with surface fish (Fig. 2e), which may reflect a potential compensatory mechanism or dysregulated response. To probe upstream regulation further, we detected the expression of *creb1a* and *creb1b*, which can enhance *mitf* expression through binding to the promoter region of *mitf* (Yuan et al. 2016; Wang et al. 2022), and found no significant differences between the two species (Fig. 2e).

To further understand these distinct expression patterns, we investigated the sequence variations of pigmentation genes between surface fish and cavefish. Firstly, the sequencing results identified a T-to-A mutation in exon 6 of *tyrp1a* in cavefish (supplementary fig. S4a, Supplementary Material online), a change that is consistent with the previously published sequences (CM045773.1) of *T. rosa*. Introducing a premature termination codon likely triggered the mRNA surveillance mechanism, nonsense-mediated mRNA decay pathway, leading to mRNA degradation (supplementary fig. S4b, Supplementary Material online). This finding was corroborated by qPCR results.

**Fig. 3.**
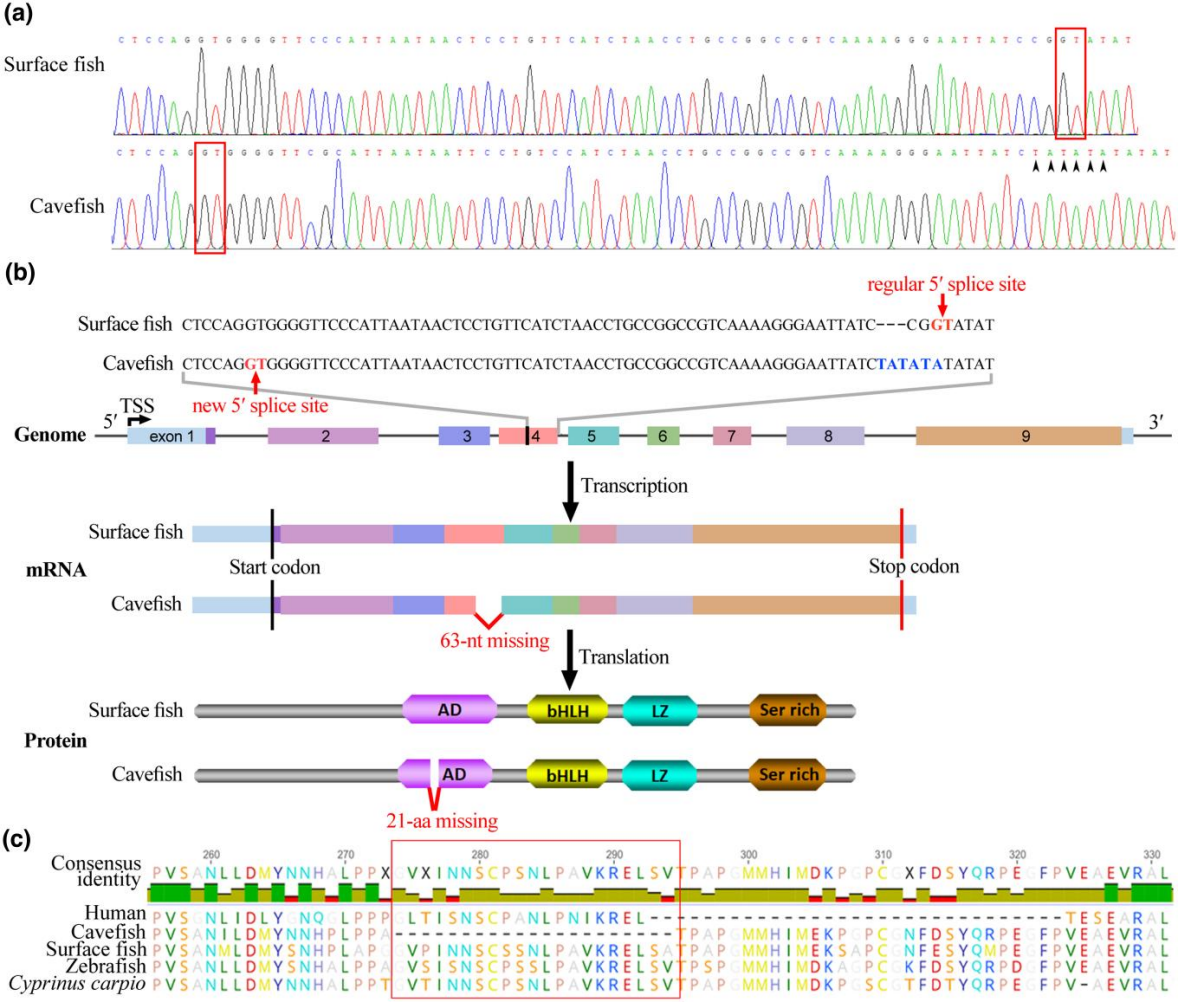
a) Sequencing chromatograms of *mitfa* in surface fish and cavefish. Base changes are indicated by black arrowheads. The GT sequence within the box in surface fish represents the canonical 5′ splice site of intron 4, while the red box in cavefish indicates the alternative splice site located within exon 4. The GC sequence within red box represents the 5′ splice site of intron 4 of *mitfa*. b) Schematic representation of *mitfa* gene structure, mRNA isoforms and protein isoforms in surface fish and cavefish. The exon/intron organization is shown, with black and red vertical lines marking the start and stop codons, respectively. In cavefish, the use of an alternative 5′ splice site leads to the loss of 63-nt in the mRNA, resulting in a protein isoform missing 21 amino acids in the AD. bHLH, basic helix-loop-helix domain; LZ, leucine zipper domain; Ser-rich, serine-rich region; TSS, Transcription Start Site. c) Alignment of Mitfa protein sequences from human, cavefish, surface fish, zebrafish and *Cyprinus carpio*. The 21-missing amino acids within the red square are for the cavefish. The 21-amino acid deletion in cavefish is highlighted within the red box.

Additionally, based on comparison of the *mitfa* sequences assembled from RNA-seq reads, we discovered that the cavefish lacked a 63-nt sequence at the end of exon 4 compared with its surface-dwelling relatives. To investigate the underlying cause of this deficiency, we amplified and sequenced the genomic DNA of this region. Our findings revealed that a mutation (from CGG to TATATA) occurred at the 5′ splice site of intron 4, which caused the upstream GT located 63 nucleotides away to be recognized as a new splice site, resulting in a shortened transcript (Fig. 3a and b). This genomic variation is consistent with the published genome (CM045779.1) of *T. rosa*. This truncated *mitfa* transcript encodes a protein with a 21-amino-acid deletion in the activation domain (AD), which is highly conserved among species; this potentially impairs its ability to activate downstream target genes (Fig. 3b and c). Together, these findings indicate that the Mitfa protein, which lacks a critical segment of its AD, may be compromised in its capacity to regulate its target genes, contributing to the observed pigmentation loss in cavefish.

### Splicing Mutation of *mitfa* Leads to Melanin Synthesis Failure

**Fig. 4.**
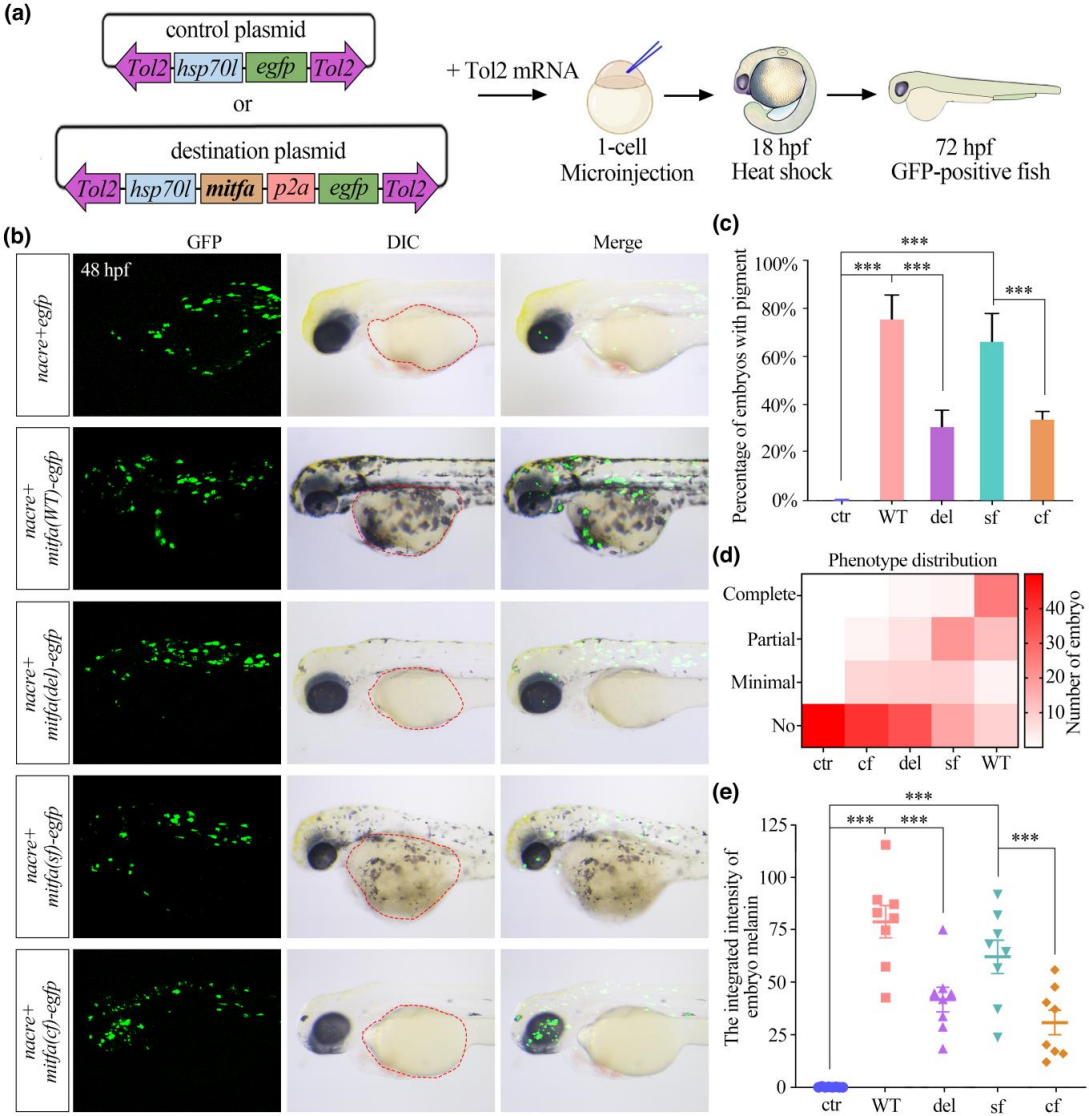
Functional validation of Mitfa variations in the zebrafish pigmentation. a) Schematic representation of the Tol2 expression constructs used to rescue the *nacre* mutant zebrafish. The control plasmid contains the *hsp70l* promoter driving *egfp* expression. The destination plasmids express *mitfa* and *egfp* independently due to the P2A “ribosome skipping” sequence. b) Representative bright-field and fluorescence images showing the melanin-enriched area of zebrafish embryos at 48 hpf in the control and four injected groups. Dashed outlines indicate the defined regions of interest used for melanin quantification. sf, surface fish; cf, cavefish; ctr, control (*egfp*-only vector without mitfa); WT, zebrafish WT *mitfa* cds; del, zebrafish *mitfa* cds carrying the 63-nt deletion identified in cavefish. All panels display lateral views (scale bar, 200 μm). c) Percentage of embryos displaying pigmentation in the control group and the four *mitfa* cds-injected groups. Data are presented as mean ± SD from three independent experiments. “***” indicates that *P* < 0.001. d) Heatmap illustrating the distribution of pigmentation phenotypes (complete, partial, minimal, or no pigmentation) across the control and injected groups (*n* = 50 per group). e) Integrated melanin intensity in lateral views of zebrafish embryos, as quantified in b). Data are presented as mean ± SD (*n* = 8 per group). “***” indicates that *P* < 0.001.

To assess whether the sequence mutation in *mitfa* contributes to depigmentation in cavefish, first, we analyzed the conservation of the AD sequence of *mitfa* with Clustal Omega and found that the 21 amino acids in the AD of surface fish Mitfa were relatively conserved in zebrafish and common carp (Fig. 3c). To functionally assess Mitfa, we performed rescue experiments in the zebrafish *nacre* mutant, which harbors a mutation in the *mitfa* gene that leads to the loss of functional Mitfa and the consequent absence of melanophores (Lister et al. 1999). One control plasmid and four experimental plasmids were successfully constructed, each containing the *hsp70l* promoter driving *egfp* or different *mitfa* alleles, including the surface fish *mitfa*, cavefish *mitfa*, zebrafish wild-type (WT) *mitfa*, and zebrafish *mitfa* with 63-nt deletion identified in cavefish (Fig. 4a). Following plasmid injection into *nacre* embryos, we observed significant green fluorescence in most injected embryos, confirming efficient plasmid uptake (Fig. 4b). By 48 h post-fertilization (hpf), embryos injected with each type of *mitfa* displayed varying degrees of melanin synthesis compared with control-injected siblings (Fig. 4b and c, supplementary table S5, Supplementary Material online). Notably, embryos injected with the zebrafish full-length *mitfa* or the surface fish *mitfa* exhibited significantly larger melanized areas on their surface than those injected with cavefish *mitfa* or the zebrafish 63-nt deletion *mitfa* (Fig. 4d and supplementary table S6, Supplementary Material online).

Quantitative analysis indicated that the highest level of melanin was observed in embryos injected with the zebrafish WT *mitfa*, followed by the surface fish *mitfa*, then the zebrafish 63-nt deletion *mitfa*, and finally the cavefish *mitfa*, which resulted in very few melanocytes (Fig. 4e and supplementary table S7, Supplementary Material online). These findings indicate that Mitfa from surface fish, rather than from cavefish, efficiently promotes melanin biosynthesis in zebrafish. Furthermore, the 63-nt deletion in cavefish *mitfa*, resulting from a splice site mutation, likely contributes to their depigmented skin phenotype observed in cavefish.

## Discussion

### Genetic Factors of Depigmentation in *T. rosa*

This study identifies major genetic mechanisms underlying pigmentation loss in the cavefish, *T. rosa*. Gene expression analysis through comparative transcriptomics between *T. rosa* and its closely related surface-dwelling species *T. stenura* revealed a significant down-regulation of critical melanogenesis genes (*oca2*, *slc45a2*, *tyr*, *tyrp1*, *mc1r*) in cavefish. Additionally, GO enrichment analysis identified an overrepresentation of terms related to pigmentation in down-regulated genes, which indicates that these molecular changes are crucial for depigmentation.

Further sequence variant analysis revealed two key mutations: a premature stop codon in *tyrp1a* and a 63-nt deletion in *mitfa*. The premature stop codon of *T. rosa tyrp1a* caused by a T-to-A mutation likely results in the *tyrp1a* mRNA that is degraded through the nonsense-mediated mRNA decay pathway (Ma et al. 2024), leading to extremely low expression levels of *tyrp1a* in *T. rosa*. In humans, mutations in *Tyrp1* lead to *oculocutaneous* albinism type 3 (Wagatsuma et al. 2023). In contrast, zebrafish possess two paralogs, *tyrp1a* and *tyrp1b*, due to teleost genome duplication. Studies have shown that *tyrp1a* mutant zebrafish still produce melanin, albeit with a reduced amount of melanin synthesis (Krauss et al. 2014).

Disruptions in *mitf* genes lead to reduced melanophore numbers and pigmentation in various species, including tilapia, humans, mice, dogs, and Peking ducks (Hodgkinson et al. 1993; Baranowska et al. 2014; George et al. 2016; Zhou et al. 2018; Wang et al. 2023a). The 63-nt deletion in *mitfa* is a critical contributor to depigmentation phenotype in *T. rosa*; this was confirmed through functional assays in *nacre* zebrafish embryos, highlighting their impact on melanin synthesis pathways. Together, these findings highlight the molecular basis of depigmentation and provide valuable insights into the evolution of troglomorphic traits in cavefish *T. rosa*.

### Evolutionary Mechanisms: Relaxed Purifying Selection and Genetic Drift

Pigmentation loss in cavefish is an exemplary model for studying regressive trait evolution. Skin color is primarily determined by melanin synthesis, which increases under UV exposure to protect the skin, and contributes to camouflage and social signaling in fish. However, these functions become unnecessary in the perpetual darkness of cave environments (Bussotti et al. 2018).

Our evolutionary analyses revealed that genes primarily involved in pigmentation in *T. rosa* branch exhibited higher Ka/Ks ratios compared with the corresponding branch in the surface fish. This indicates relaxed purifying selection on pigmentation-specific genes in the cave environment (Fig. 2a). Interestingly, although pigmentation-specific genes also exhibited slightly higher *ω* than pigmentation-related genes in the surface fish lineage (0.251 vs. 0.186, *P* = 0.02), this difference was even more pronounced in the cavefish lineage, where pigmentation-specific genes displayed a substantially higher mean *ω* and a much stronger statistical significance (0.547 vs. 0.246, *P* = 0.00056). Meanwhile, multifunctional pigmentation-related genes consistently showed the lowest *ω* in both lineages, likely because of their involvement in a wide range of biological processes and their maintenance under stronger purifying selection. Additionally, we found that both the median Ka and Ks values were lower in the cavefish *T. rosa* compared with the surface fish (supplementary fig. S5, Supplementary Material online). This suggests that cavefish may have experienced reduced substitution rates, potentially due to factors such as potentially longer generation times, lower metabolic rates, and the absence of UV exposure in cave environments. This trend is similar to the reduced mutation rates observed in deep-sea fishes (Xu et al. 2025).

In subterranean habitats where pigmentation provides no adaptive advantage, mutations in pigmentation genes that are deleterious in surface environments may become effectively neutral once purifying selection is relaxed, and may then be fixed by genetic drift (Horst and Ulrike 2017). Additionally, in small and isolated cavefish populations, genetic drift is further enhanced, further contributing to the widespread loss of pigmentation observed in *T. rosa* and other independently evolved cavefish lineages. It is important to note that gene decay in the process of regressive evolution may occur gradually along the evolutionary lineage through relaxation of selective constraints at specific sites within genes (Meredith et al. 2009). Future studies incorporating additional cave-dwelling *Triplophysa* genomes may enable us to test this gradual relaxation model of pigmentation gene evolution in cave environments.

Given that cis-regulatory changes in *mc1r* have also been implicated in regressive pigmentation in cavefish, they may function alongside coding mutations in contributing to pigmentation loss (Gross et al. 2016). Moreover, regulatory elements of pleiotropic genes controlling pigment cell-specific expression may face relaxed selection in cave environments, even when their coding regions remain conserved. Further studies are needed to clarify the relative contributions of coding and regulatory mutations to pigmentation loss.

Prior studies on *A. mexicanus* have shown that mutations in *oca2* disrupt upstream steps in melanin synthesis, contributing to albinism. This indicates a potential adaptive role for pigmentation loss, possibly linked to catecholamine-related processes that influence behavioral or physiological traits in cave environments (Bilandžija et al. 2018). In our study, comparative protein sequence analysis identified multiple amino acid alterations in Oca2 in *T. rosa* (supplementary fig. S6, Supplementary Material online), which may similarly impact melanin synthesis pathways. Although our current study lacks direct evidence of natural selection acting on pigmentation loss in *T. rosa*, it is still a possibility worth exploring. Some studies have suggested that the initial loss of pigmentation might confer an adaptive advantage by reallocating energy to other processes in nutrient-scarce cave environments (Culver and Pipan 2019). However, subsequent mutations in other pigmentation genes are more likely to have accumulated as a result of relaxed purifying selection, and to have become fixed by genetic drift, rather than under sustained positive selection. This highlights that, while positive selection may have contributed initially, relaxed selection likely predominates in shaping the overall pattern of pigmentation gene decay in cavefish.

In light of these findings, we also estimated the cave settlement time of *T. rosa* to be ∼5.37 Ma. Although *mitfa* and *tyrp1a* carry confirmed LoF mutations, most other pigmentation-specific genes may still be under selection, even if partially relaxed. In such cases, the inferred timing should be considered an upper bound for the age of cave settlement, suggesting that the cave settlement of *T. rosa* may be older than this estimate. Broader genomic sampling and more precise understanding of mutation rates in the *Triplophysa* lineage will be essential for improving the accuracy of this estimate.

### Alternative Splicing as a Molecular Mechanism in Pigmentation Loss

A particularly novel finding of our study is the role of alternative splicing in the evolution of pigmentation loss. The 63-nt truncation in *mitfa* in *T. rosa*, caused by the nucleotide mutations at the 5′ splice site that introduce a novel splicing event, significantly disrupts melanogenesis. Alternative splicing has long been recognized as a driver of phenotypic diversity and rapid adaptation. Our results extend this understanding to the context of regressive evolution, suggesting that splicing mutations can contribute to trait regression in cave-dwelling species.

Future research should focus on exploring additional genes and pathways involved in pigmentation, assessing the functional impacts of these genetic changes, refining the temporal and evolutionary dynamics of pigmentation gene decay using more cavefish genomic data, and investigating the relative contributions of coding and regulatory mutations. These efforts will further illuminate the complexities of pigmentation loss and clarify the evolutionary drivers of this trait in extreme environments.

## Materials and Methods

### Fish Species Collection

The surface fish, *Triplophysa stenura* was collected from Kangding County, Sichuan Province, while the cavefish, *Triplophysa rosa* was collected from Huolu Town, Wulong County, Chongqing City (supplementary fig. S7, Supplementary Material online). Adult zebrafish of the WT AB strain, and *nacre* (*mitfa^w2/w2^*), sourced from the China Zebrafish Resource Center (CZRC ID: CZ1451), were maintained in system water at 28 ± 0.5 °C under standard conditions. Zebrafish embryos were obtained through natural spawning and incubated at 28 °C. All experimental procedures involving animals were conducted and approved by the Animal Care and Use Committee of the Institute of Zoology, Chinese Academy of Sciences (IOZ18002).

### H&E Staining and Transmission Electron Microscopy

For H&E staining, adult *Triplophysa* fishes were euthanized, and lateral skin samples with partial muscle were taken from the trunk region between the head and dorsal fin. Skin samples were fixed overnight in 4% paraformaldehyde in phosphate buffered saline at 4 °C, followed by equilibration in 30% sucrose prior to cross-cryosectioning (Leica CM3050S, Leica Biosystems). Slides were stained with H&E using standard procedures.

For transmission electron microscopy, skin samples from different fish were fixed and sectioned as previously described (Ullate-Agote et al. 2020). Ultrathin sections (70 nm thick) were obtained using a microtome (Leica EM UC6) and double-stained with uranyl acetate and lead citrate. Micrographs were captured with a transmission electron microscope (Hitachi TM-1000, Center for Biological Imaging, Institute of Biophysics, Chinese Academy of Sciences) at an acceleration voltage of 120 kV. Large montage alignments were carried out utilizing the Blendmont command-line from IMOD software. Display levels of the images were adjusted with ImageJ to facilitate comparisons.

### Construction of the Pigmentation Gene Set

The pigmentation gene set in this study was constructed to include genes involved in pigmentation processes, melanocyte development and differentiation, and melanosome biology. In line with the objectives of this study, genes specifically related to iridophores, xanthophores, or the RPE were excluded. To ensure data consistency and comparability, the initial gene filtering was restricted to *D. rerio* (Taxonomy ID: 7955), selected as the representative teleost species. We compiled the initial pigmentation gene list by identifying *D. rerio* genes annotated with relevant GO terms from the GO database (https://www.geneontology.org/). The GO terms included: “pigmentation” (GO:0043473), “pigment accumulation” (GO:0043476), “melanosome transport” (GO:0032402), “melanosome organization” (GO:0032438), “melanocyte differentiation” (GO:0030318), and “developmental pigmentation” (GO:0048066). We then manually curated the list of automatically annotated pigmentation genes by removing unnamed or uncharacterized entries as well as redundant genes assigned different GO categories, yielding a final nonredundant set of 115 unique genes (see supplementary table S2, Supplementary Material online).

Subsequently, we subdivided these pigmentation genes into two categories: pigmentation-specific genes and pigmentation-related genes. Pigmentation-specific genes were defined as those primarily involved in melanin synthesis, melanosome formation, or pigment cell differentiation. These genes are typically supported by experimental evidence, such as knockout or knockdown studies showing pigmentation defects, or are known to be associated with pigmentation disorders in humans or model organisms. In contrast, pigmentation-related genes were identified based on literature and/or knockout studies as having pleiotropic functions or broad expression patterns. Although not exclusively associated with pigmentation, these genes may influence melanocyte development or pigmentation indirectly through roles in developmental or signaling pathways. The classification of genes into these two subcategories is indicated in the second column (Classification of Pigmentation Genes) of supplementary table S2, Supplementary material online, with labels “specific” or “related”.

### Transcriptome Sequencing

Surface fish and cavefish were euthanized immediately after field collection, and skin samples were preserved in RNAlater (Ambion, USA). Total RNA was extracted from the skin samples of the both species (*n* = 3 per species) using TRIzol reagent (Invitrogen, Carlsbad, CA, USA). After selection with oligo(dT) magnetic beads, transcriptome libraries of the skin from both *Triplophysa* species were constructed following the manufacturer's protocol. Libraries of 200 to 400 bp inserts were sequenced on an Illumina Hiseq 2000 instrument (Illumina, San Diego, CA, USA) to produce paired-end 150-nucleotide reads. Raw reads were cleaned by removing adaptor sequences, poly-N and low-quality reads using Fastp.

### Calculating Selective Pressure on Pigment Genes

The Ka/Ks analysis allowed us to estimate lineage-specific Ka/Ks values for the *T. rosa* and *T. stenura* branches, thereby assessing selective pressures acting on pigmentation genes in the cavefish lineage.

We downloaded the coding sequences (CDS) of *T. rosa* and *D. rerio* (GRCz11), and obtained the assembled transcripts of *T. stenura* using Trinity v2.1.1 with default parameters (Grabherr et al. 2011). Orthologous genes among the three species were identified through the PosiGene (v0.1) with default settings (Sahm et al. 2017). Then we used the codeml program of the PAML software package (v4.9) (Yang 2007) to estimate the Ka/Ks ratios (*ω*) for orthologs along different evolutionary branches, incorporating *D. rerio* as an outgroup. We applied the branch model (model = 2) to estimate lineage-specific *ω* for the *T. rosa* and *T. stenura* branches, thereby assessing the selective pressures acting on pigmentation genes in the cavefish lineage. Subsequently, we generated Ka/Ks distribution plots for all orthologous and candidate gene sets with R package ggplot2, comparing the Ka/Ks values of the cavefish and surface fish branches individually, applying the wilcox.test to assess the significance of differences between lineages.

### Estimating the Period of Neutral Evolution of Pigmentation-specific Genes in *T. rosa*

Pigmentation-specific genes from *T. rosa*, *T. stenura*, and the outgroup species (*D. rerio*) were concatenated and aligned. We assumed that these pigmentation-specific genes have been subject to purifying selection along the branches of the surface-dwelling lineage, while the branch leading to *T. rosa* represents a mixed branch—initially under purifying selection, followed by a period of complete relaxation of selection after settlement in the cave environment. We estimated the time since purifying selection was relaxed in the cavefish branch using the framework described in previous studies (Meredith et al. 2009; Policarpo et al. 2021). The estimated divergence time between *D. rerio* and *Triplophysa* is ∼97 million years ago (Ma) (http://www.timetree.org/). The calculation details and the equation used for the mixed branch are provided in our GitHub repository. https://github.com/cavefish-fanwei/Depigmentation-in-cavefish-Triplophysa-rosa.

### Differential Expression, GO, and Pathway Enrichment Analysis

To quantify the gene expression levels of two *Triplophysa* species, we used downloaded CDS sequences of *T. rosa* and the assembled transcripts of *T. stenura* as the reference transcriptome for each species. Clean reads were mapped to their respective reference transcriptomes with Bowtie 2 (2.5.1) (Langmead and Salzberg 2012) with default settings. Mapped reads were converted to TPM values to quantify transcript expression. DEGs were identified using the DEseq2 R-packages (Love et al. 2014), with a threshold of |fold change| > 2 and adjusted *P* < 0.05.

Analyses of GO and KEGG pathway enrichment of the DEGs were performed by KOBAS 3.0 and DAVID online tools (Bu et al. 2021; Sherman et al. 2022). Results from the GO functional enrichment analysis were categorized into biological process, cellular component, and molecular function. GSEA was carried out by searching KEGG Database (Subramanian et al. 2005). The GO categories and KEGG pathways with *P* < 0.1 were selected as the cut-off criterion.

### Quantitative Real-Time Polymerase Chain Reaction

cDNA samples were synthesized from total skin RNAs using a First-Strand cDNA Synthesis Kit (Invitrogen, Carlsbad, CA, USA). Real-time PCR was performed with the CFX96 Real-Time PCR system (Bio-Rad, Singapore) using SYBR Green (TaKaRa, Dalian, China). Primer sequences are list in the supplementary table S8, Supplementary Material online. The relative gene expression levels were normalized to the expression of *β-actin*, applying the relative Ct method. Three biological replicates were used for each gene. Statistical significance was assessed with a two-tailed Student's t-test in Microsoft Excel with *P* < 0.05.

### Sequencing of *mitfa* and *tyrp1a* Genomic Regions in Surface Fish and Cavefish

Genomic DNA samples of *T. rosa and T. stenura* were extracted from fin clips using marine animals DNA kit (Tiangen Biotech, Beijing, China). Target genomic regions were amplified using the following primers: *mitfa-g-F, mitfa-g-R, tyrp1a-cf-g-F, tyrp1a-cf-g-R, tyrp1a-sf-g-F*, and *tyrp1a-sf-g-R* (see supplementary table S8 for all primer sequences), which were designed based on the CDS sequences of *Triplophysa mitfa* and *tyrp1a*.

### Making Constructs and Embryonic Overexpression Assay

We selected the zebrafish model system to investigate the functional role of *mitfa* in melanin synthesis in both surface and cave *Triplophysa* species. Firstly, we constructed four plasmids, each with *hsp70 l* promoter driving the CDS of *mitfa* with different genotypes. These *mitfa* genotypes included the surface fish, cavefish, zebrafish, and a zebrafish *mitfa* containing the 63-nt deletion identified in cavefish (denoted as zebrafish *mitfa* deletion). All plasmids were validated by sequencing. Downstream of the *mitfa* gene, an enhanced green fluorescent protein (eGFP) was linked via P2A, allowing for easy identification of transgenic embryos. Second, *nacre* embryos were co-injected with premixed *Tol2* mRNA and the same amount one of the above plasmids (50 pg plasmid and 100 pg *Tol2* mRNA per embryo) at the one-cell stage. Embryos were then heat-shocked at 37 °C for 1 h at 16 h post-fertilization (hpf). Following heat shock, successful induction of transgene expression was confirmed by observing bright green fluorescence in the injected embryos. Only embryos displaying this fluorescence were collected for further analysis, and their bright-field and fluorescence images were captured using the laser scanning confocal microscopy (Nikon, Japan). Statistical significance was assessed with a two-tailed Student's *t*-test in Microsoft Excel with *P* < 0.001.

## Supplementary Material

msaf175_Supplementary_Data

## Data Availability

All sequencing data generated for this project is available under CRA006704. All custom scripts and configuration files used in this article can be found in: https://github.com/cavefish-fanwei/Depigmentation-in-cavefish-Triplophysa-rosa.
