# Supplementary material for: A Splicing Mutation in mitfa is Involved in the Depigmentation of Cavefish Triplophysa rosa
Source: Mol Biol Evol. 2025 Jul 25;42(8):msaf175. doi: 10.1093/molbev/msaf175 (PMC12343024; doi:10.1093/molbev/msaf175)
Supplement: msaf175_Supplementary_Data [file msaf175_supplementary_data.zip › Supplementary materials-MBE-25-0024.pdf]

SUPPLEMENTARY MATERIALS

A Splicing Mutation in *mitfa* is Involved in the Depigmentation of Cavefish *Triplophysa rosa*

Mingming Zhang[1#], Qingshuo Zhao[1#], Jinqing Huang[2#], Ming Zou[3], Baocheng Guo[1§] and Yahui Zhao[1§], Fanwei Meng[1§]

1. State Key Laboratory of Animal Biodiversity Conservation and Integrated Pest Management, and Key Laboratory of Zoological Systematics and Evolution, Institute of Zoology, Chinese Academy of Science, Beijing, 100101, China.
2. Guilin Medical University, Guilin, Guangxi, 541199, China.
3. School of Nursing and Health Management, Wuhan Donghu College, Wuhan, Hubei, 430212, China.

[#] These authors contributed equally to this work

[§] Corresponding author: Fanwei Meng, E-mail: mengfw@ioz.ac.cn;

co-corresponding author: Yahui Zhao, E-mail: zhaoyh@ioz.ac.cn;

co-corresponding author: Baocheng Guo, E-mail: guobaocheng@ioz.ac.cn.

The supplementary file includes:

Tables S1, S5 to S8

Figures S1 to S7

Table S1. Summary of sequencing data

Sample	Raw Reads	Clean Reads	Clean Bases	Q20	Q30	GC Content
*T. stenura*_1	47759654	47521498	7.13 Gb	97%	92%	47.16%
*T. stenura*_2	58006524	57782468	8.67 Gb	97.7%	93.5%	47.54%
*T. stenura*_3	58236944	58047510	8.71 Gb	97.5%	92.9%	46.21%
*T. rosa*_1	36127906	35362042	5.3 Gb	100%	100%	47.73%
*T. rosa*_2	36018388	35252320	5.29 Gb	100%	100%	46.63%
*T. rosa*_3	35818820	35077104	5.26 Gb	100%	100%	47.36%

Table S5. The statistical data of rescue experiment

	Phenotype of embryo	ctr	WT	del	sf	cf
The first experiment	Rescued	0	293	165	71	95
	No rescued	0	38	201	15	141
The second experiment	Rescued	0	52	32	15	45
	No rescued	0	10	109	19	109
The third experiment	Rescued	0	18	25	35	25
	No rescued	0	14	70	12	48

Table S6. The statistical data of phenotype distrbution

Phenotype distrbution	ctr	WT	del	sf	cf
Complete	0	26	4	3	0
Partial	0	13	5	20	2
Minimal	0	2	9	13	8
No	50	9	32	14	40
Sum	50	50	50	50	50

Table S7. The integrated intensity of embryo melanin

Embryo number	ctr	WT	del	sf	cf
1	0	117.874	77.388	75.308	22.653
2	0	89.585	18.449	66.924	39.561
3	0	77.195	47.147	94.175	50.215
4	0	85.505	46.924	26.237	18.449
5	0	91.735	20.758	70.537	42.803
6	0	45.123	44.334	84.329	19.572
7	0	82.999	31.201	59.225	58.264
8	0	59.783	36.235	39.51	14.473

Table S8. List of primers

Primer	5′-3′	Use
creb1a-F	CCAGCAATCAAGTAGTAGTG	qPCR
creb1a-R	GCCTCCCTGTTCTTCATAA	qPCR
creb1b-F	AAGCAATCAGGTGGTTGTA	qPCR
creb1b-R	CTCCAGACACTTGACATATTC	qPCR
mc1r-F	GTCTGACATGCTGGTGAG	qPCR
mc1r-R	GATGCTGTGGTAGCGAAG	qPCR
mitfa-F	AGACATGAGGTGGAATAAGG	qPCR
mitfa-R	GCTACAAGGTCAGAGGAATA	qPCR
oca2-F	AGTCCTCCTGGTCAAGTG	qPCR
oca2-R	AGCAGCGAAGAACAACAA	qPCR
pmela-F	ACTCTGGCACCTTCTGTAT	qPCR
pmela-R	TTCCTGTAATCTGCTCTCAC	qPCR
pmelb-F	GGAGATGGAGCGTAACATT	qPCR
pmelb-R	AGAGTGCCTGAGGATGAG	qPCR
slc45a2-F	TCCTATGCTACTTACGAGAG	qPCR
slc45a2-R	TTCTCTGCTCCTCTTCCA	qPCR
tyr-F	GCACGGATGAACTGATGG	qPCR
tyr-R	CGGTCTCGTACTCTGTTAG	qPCR
tyrp1a-F	GAGAGCGTGGAGGAGTAT	qPCR
tyrp1a-R	CCACTGGATCATAATTCCCC	qPCR
tyrp1b-F	CGAGAGTGTGGAGGAGTA	qPCR
tyrp1b-R	TCTGAGAGGAAGTGGAGTAA	qPCR
β-actin-F	GAAGATCAAGATCATTGCTCCC	qPCR
β-actin-R	ATGTCATCTTGTTCGAGAGGT	qPCR
mitfa-g-F	TGGACGATGTAATTGAAGAC	genotyping
mitfa-g-R	CTTCTCCATTATGTGCATCA	genotyping
tyr1a-sf-g-F	GCCCACCTGTTTCTAAAC	genotyping
tyr1a-sf-g-R	TCGTAGCGTTGATAATGG	genotyping
tyr1a-cf-g-F	GCCCACCTGTTTCTGAAC	genotyping
tyr1a-cf-g-R	TCGTAGCGTTGGTAATGC	genotyping

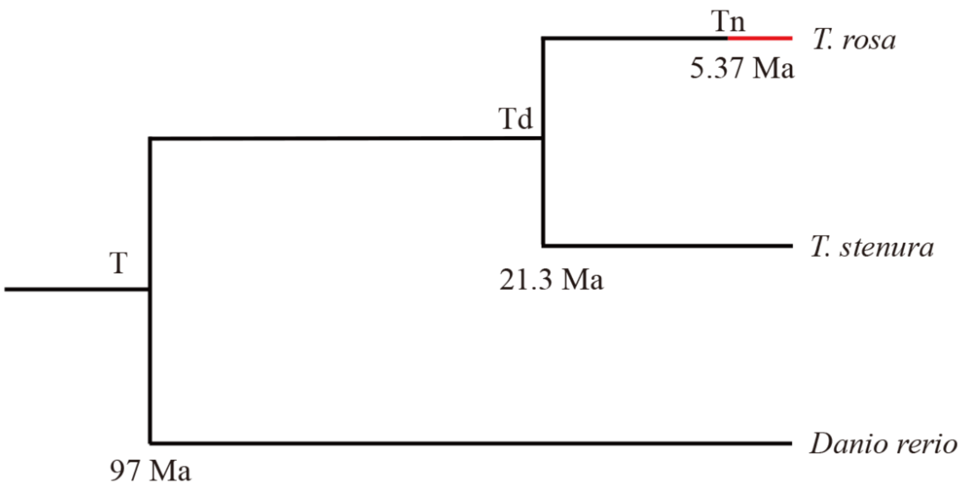

Fig. S1. Estimates of the period of neutral evolution of *T. rosa* pigmentation-specific genes. T, divergence time of *D. rerio* and *Triplophysa*; Td, divergence time of *T. stenura* and *T. rosa*; Tn, period of neutral evolution; Ma, million years ago.

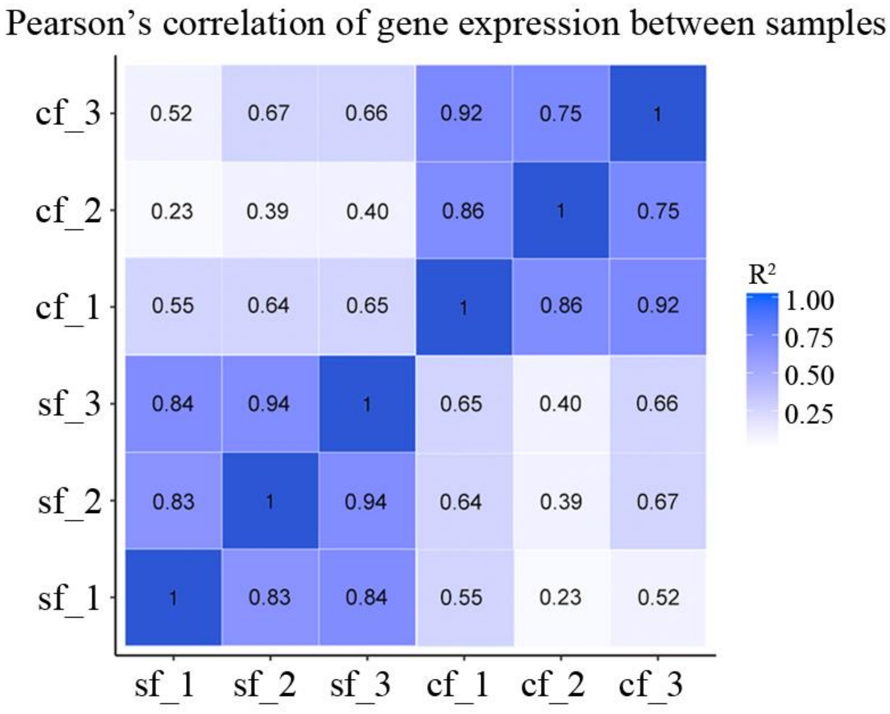

Fig. S2. Heatmap showing Pearson's correlation of gene expression between samples. The x-axis and y-axis represent individual samples. Blue indicates a strong correlation, while white represents a weaker correlation. sf, surface fish; cf, cavefish.

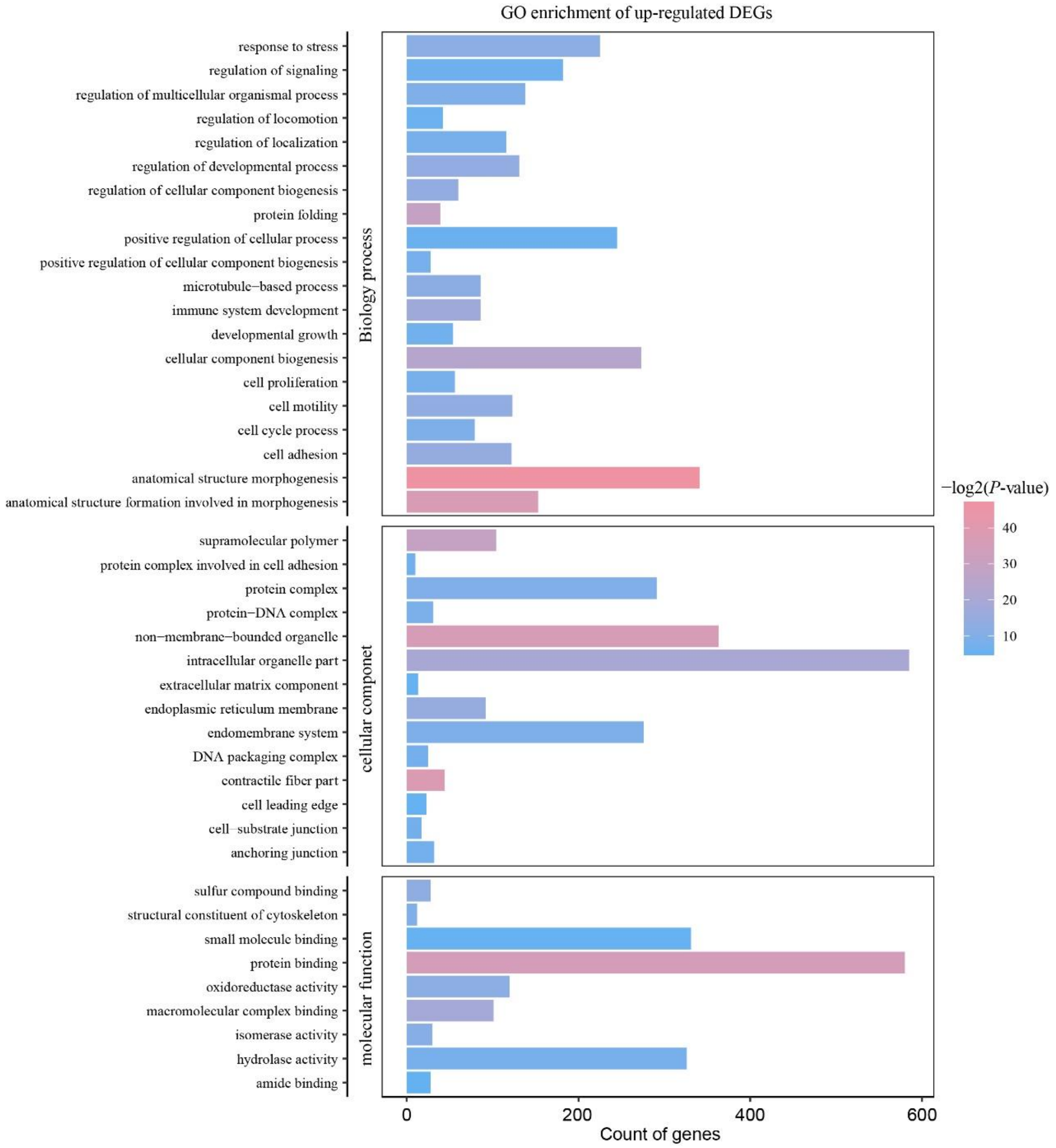

Fig. S3. GO enrichment analysis of up-regulated DEGs in cavefish across three main categories. The x-axis represents the numbers of genes associated with each GO term, while the y-axis lists the GO terms grouped into three categories. Colors indicate the corresponding *P*-value.

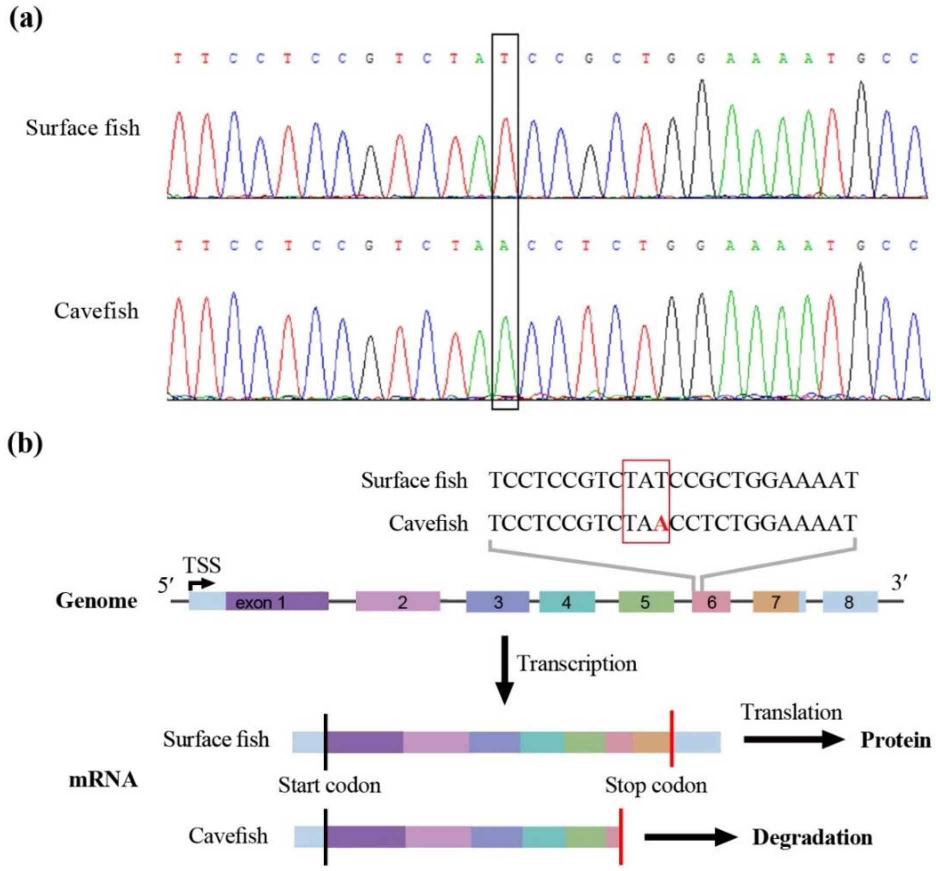

Fig. S4. Analysis of the *tyrp1a* gene and its mRNA isoforms in surface fish and cavefish. (a) Sequencing chromatograms showing the base peaks of *tyrp1a* in surface fish and cavefish. The base changes are denoted by black box. (b) Schematic representation of *tyrp1a* gene structure and its corresponding mRNA isoforms in surface fish and cavefish. The distribution of exons and introns, along with the mRNA isoforms, is illustrated. Black and red vertical lines indicate the positions of the start codon and stop codon, respectively. TSS: Transcription Start Site.

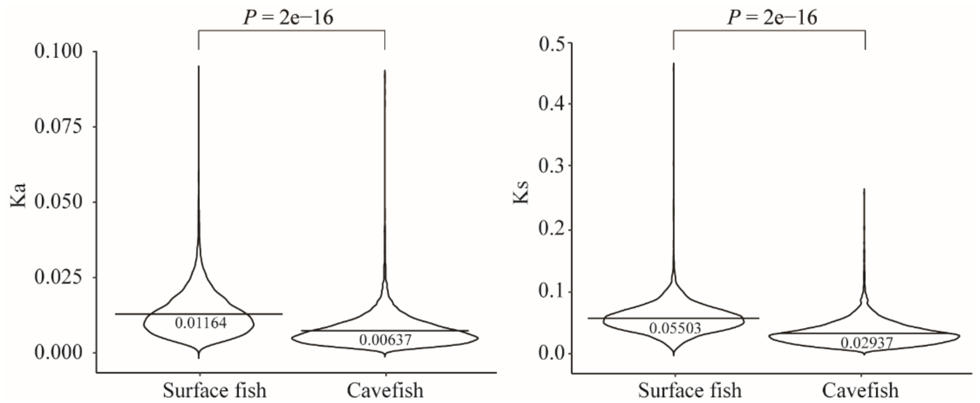

Fig. S5. Distributions of Ka and Ks across species. Violin plots illustrating the Ka and Ks distributions in the two species, with the median values indicated. Statistical differences between groups were assessed, and *P*-values are shown above the corresponding horizontal bars.

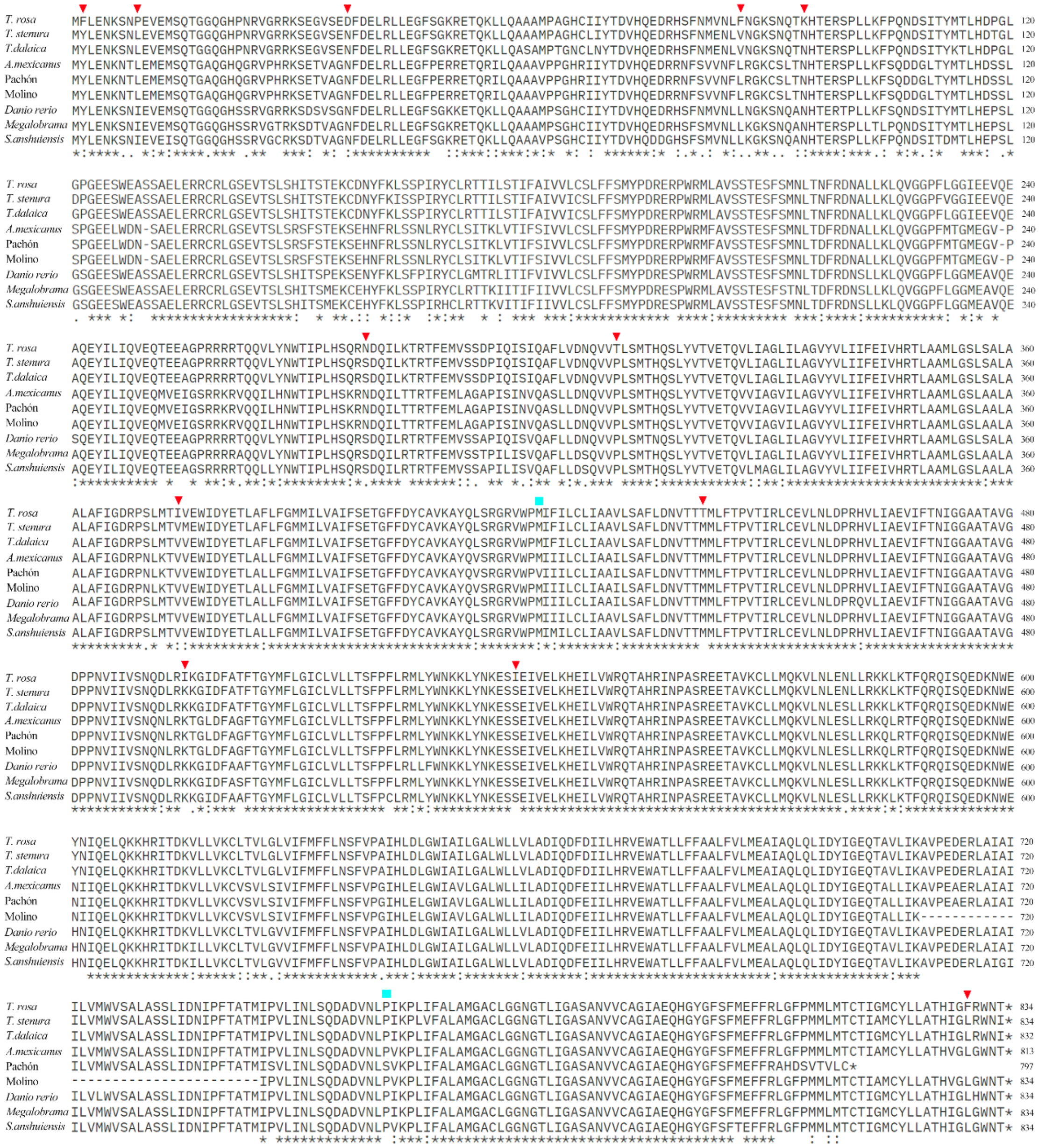

Fig. S6. Alignment of Oca2 protein sequences in *T. rosa*, *T. stenura*, *T. dalaica*, *Danio rerio*, *A. mexicanus*, *Megalobrama* and *S. anshuiensis*. The red triangles indicate the amino acid changes in *T. rosa* compared to *T. stenura*. The turquoise squares indicate the amino acid changes in the Pach ón (cave population) compared to the *A. mexicanus* (surface population). '*' indicates positions with a single identical residue; ':' indicates that the amino acid at that position is weakly conserved; '.' indicates that the amino acid at that position is strongly conserved.

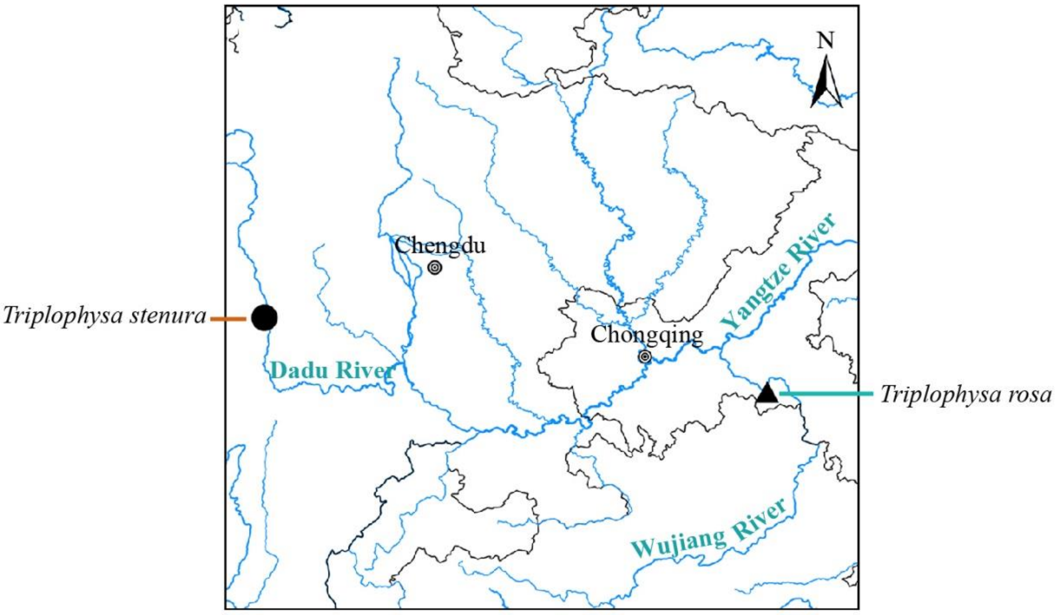

Fig. S7. Map showing collection localities of *Triplophysa rosa* (black triangle) and *Triplophysa stenura* (black circle) within their respective habitats.
